# Systemic Barriers to Curriculum Adaptation for Rapidly Changing Knowledge in Medical Education: Qualitative Study

**DOI:** 10.2196/96244

**Published:** 2026-07-15

**Authors:** Martin Baumgartner, Matthias Stadler, Matthias Samwald, Martin R Fischer

**Affiliations:** 1 Teaching Center, Medical University of Vienna, Spitalgasse 23, Vienna, 1090, Austria, 43 6647632200; 2 Institute of Medical Education, LMU University Hospital, LMU Munich, Munich, Germany; 3 Institute of Artificial Intelligence, Center for Medical Data Science, Medical University of Vienna, Vienna, Austria

**Keywords:** curriculum, artificial intelligence, program development, digitalization, curriculum governance, medical education

## Abstract

**Background:**

For this study, digitalization in medicine was used as an illustrative case to investigate how medical curricula respond to rapidly changing knowledge. Digitalization is transforming the way medicine and health care are provided and experienced. Experts have suggested various topics for medical curricula to keep pace with rapidly evolving knowledge. However, adapting these curricula remains a lengthy process that often lacks an interdisciplinary approach.

**Objective:**

The perspectives of curriculum governing bodies and the boards responsible for curriculum operations at 2 medical universities were examined regarding the need for curriculum changes to account for digitalization in medicine, as well as the difficulties in adapting the curriculum to ever-growing knowledge. We identify and suggest ways to achieve more agile curriculum development.

**Methods:**

This study consists of a qualitative analysis of governing policy frameworks and a qualitative study involving 14 video interviews. The interviews were performed with members of university curriculum governing bodies and the boards responsible for curriculum operations.

**Results:**

All participants agreed that digitalization will reshape the medical profession by reducing physical contact, enhancing data-driven communication, and streamlining administrative processes. They highlighted the need for graduates to acquire digital literacy, critical evaluation skills, and a basic understanding of data and statistics. Yet, despite being designed as an integrated program, most participants noted that curricula have become fragmented over time due to a lack of coordination between curriculum modules. Furthermore, current processes lead to a siloed perspective, where limited coordination between modules makes it difficult to implement new knowledge holistically. This lack of intermodule alignment emerged as a key barrier to coherent curricular change. Learning objectives were identified as a promising but underutilized tool for monitoring content, aligning modules, and ensuring that emerging topics such as digitalization are integrated consistently.

**Conclusions:**

Most participants agreed that current processes for monitoring and updating curricula are not efficiently designed, tending to be too static and focusing primarily on the advancement of subject-specific medical knowledge. To prepare current and future students for a rapidly changing world, curriculum processes should evolve from static, fragmented structures to more agile, integrated systems. By mapping the survey results to the curriculum development frameworks of Kern and Harden, we find that the challenge lies not so much in adding new content, but rather in designing curriculum processes that achieve a holistic overview. Strengthening the use of learning objectives as a dynamic monitoring and alignment tool offers a concrete opportunity to integrate rapidly changing knowledge holistically.

## Introduction

The European Commission emphasizes the pivotal role of universities in the “Europe of knowledge” [1]. However, the scientific literature on the development, monitoring, and quality assurance of curricula has so far only insufficiently addressed how quickly disruptive knowledge should be integrated into university curricula [2,3]. In the medical field, where safety and quality are of high importance, there is hardly any literature on this topic. The goal of our study was to better understand how quickly the medical curriculum can and should adapt to rapidly changing knowledge, using digitalization in medicine as an example of a presumably rapidly evolving domain [4].

This research is grounded in the curriculum development frameworks of Kern [5] and Harden [6]. Kern addresses curriculum monitoring as part of an iterative approach to curriculum development [5], which begins by identifying a health problem or societal need that the curriculum should address. In the “Goals and Objectives” step, broad educational goals and measurable learning objectives are defined. At the program level, these broad goals ensure that the various modules are aligned with overarching educational needs. The evaluation step assesses the achievement of goals and learning objectives for both students and the program itself, allowing for continuous improvement after each course iteration.

Harden [7] pioneered the concept of curriculum mapping in medical education. In his model, overarching learning outcomes serve as thematic anchors under which specific learning objectives are organized. The resulting curriculum map enables the identification of gaps and overlaps while visualizing dependencies between learning outcomes. The concepts of Kern and Harden are partly intertwined. Kern is more relevant to the development and renewal of specific courses, while Harden takes a strategic view of an entire program. Consequently, reviewing a curriculum map is a high-level strategic process that often involves a curriculum committee and longer review cycles.

Since the COVID-19 pandemic, attention to digital applications in medicine has increased exponentially, and this trend continues [8-11]. This has resulted in a growing number of publications calling for additional content within existing medical curricula [12-14]. Numerous studies focus on introducing new elective courses [15-17] to provide undergraduate medical students with digital competencies. However, because digitalization in medicine lacks a single “home” specialty that could serve as a natural anchor for structured content development, these initiatives tend to emphasize current technologies and specialty-specific applications, such as medical imaging in radiology or dermatology [18,19] or digital therapeutics in psychiatry [20]. Sumner et al [21] argue that “the lack of consensus in the literature on digital health content, skills and pedagogies for medical curricula may lead to overloaded frameworks with excessive content, hindering implementation.” A coordinated, broad foundation, including general topics such as telemedicine, electronic health records, or administrative applications such as automated history taking, is currently missing.

In addition, many of these initiatives are developed within existing, rather static curricular frameworks based on current knowledge. No general approach has been developed to ensure the long-term relevance of these curricula. In particular, the fundamental question of how quickly new, disruptive content can be identified and systematically integrated into medical curricula remains unaddressed.

Governance structures may differ across countries, but the underlying challenge of integrating fast-moving knowledge into traditionally slow-changing curricula appears to be universal. Therefore, this paper addresses 2 research questions. First, it examines the intentions of government and governing bodies regarding curriculum monitoring and the implementation of new content through a qualitative analysis of the relevant policy framework. Second, it explores current curricular adaptation practices in Austria by examining 2 medical universities. A qualitative study was performed at the largest public medical university in Austria, the Medical University of Vienna (MUW), with around 8900 students, and the smallest public medical university, the Medical University of Innsbruck (MUI), with around 3600 students. Both MUW and MUI emerged as independent universities from their respective medical faculties (at the University of Vienna and the Leopold-Franzens University of Innsbruck, respectively) in 2004.

To contextualize and interpret our findings, the results of this study were compared with the Kern 6-Step Approach to curriculum development [5] and the Harden concepts of the spiral curriculum and curriculum integration [6,22,23].

## Methods

### Method 1: Qualitative Policy Framework Analysis

To address the first research question, we identified the requirements for curriculum monitoring and change processes within the policy frameworks governing Austrian medical universities. We conducted a qualitative analysis of the most recent, publicly available higher education policy documents. The sample included national legislative frameworks retrieved from the Federal Legal Information System (RIS database), university-specific statutes, as well as quality audit reports and white papers sourced from the universities’ websites. A complete list of the included documents is provided in Table S1 in Multimedia Appendix 1.

The documents were systematically searched using the keywords “monitoring,” “quality,” “curriculum,” and “learning objectives.” Data analysis followed the qualitative content analysis framework proposed by Kuckartz and Rädiker [24]. The national policy and university-specific documents provided a foundational, saturated perspective, which was further triangulated with insights from quality reports and white papers. To ensure interpretive rigor, the analysis was performed by a researcher holding a master’s degree in business law.

### Method 2: Qualitative Study of the Curriculum Change Process

For the second research question, the COREQ (Consolidated Criteria for Reporting Qualitative Research) checklist was applied [25].

### Inclusion Criteria and Recruitment

**Figure 1.**
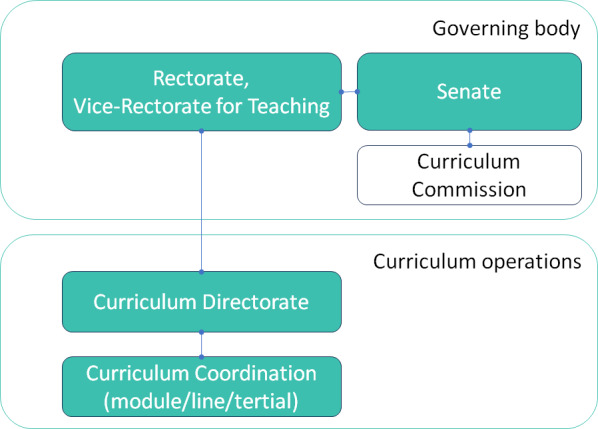
Curriculum process responsibilities at the Medical University of Vienna.

Inclusion criteria targeted academic and clinical staff from MUW and MUI with significant involvement in the planning and operation of the medical curriculum. Specifically, individuals serving on either the curriculum governing body or the curriculum operations boards were eligible for participation (Figures1  2). Other stakeholders, such as students, policymakers, and administrative staff, were excluded because they typically lacked in-depth knowledge in both areas: digitalization and curricular processes.

**Figure 2.**
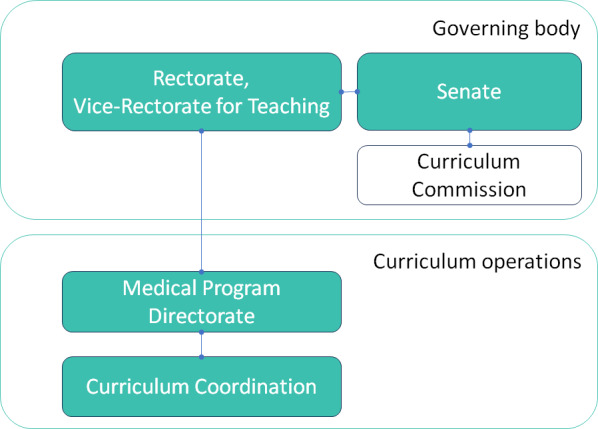
Curriculum process responsibilities at the Medical University of Innsbruck.

A modified snowball sampling approach was used for recruitment. Senior key individuals with long-standing experience in curriculum development and operations were identified and asked to nominate additional potential participants. To ensure a broad range of perspectives, the final sample was balanced across university governing bodies versus boards responsible for curriculum operations (Figures1  2), as well as preclinical versus clinical teaching. Gender was also taken into account to ensure a balanced sample.

### Study Design

A qualitative study was conducted based on semistructured interviews with the following 4 key questions:

- How are the demands placed on doctors changing due to the possibilities of digitalization?
- What are the potential future scenarios for the medical profession over the next 10 to 20 years?
- What processes and challenges are involved in updating the curriculum?
- Necessity and speed of curriculum updating: how dynamic does a curriculum need to be?

The semistructured interview guide was developed based on previous studies by Baumgartner et al [12,26] and Car et al [27] to gain a more in-depth understanding of both formal and informal processes involved in curriculum updating.

### Data Collection

Before use, the interview guide was tested with a member of the research team who serves as Vice Dean for Clinical Education in Human Medicine at a university not involved in the study. All interviews were conducted in German using the video conferencing tool Webex (version 45.9.0.33069; Cisco Systems), utilizing its built-in transcription function. Individual video meetings were scheduled between each participant and the researcher. Interviews were planned to last approximately 50 minutes, beginning with the participant’s professional profile followed by the interview questions. Data analysis followed the framework proposed by Kuckartz and Rädiker [24]. As described in the framework, data collection and data analysis are interwoven. Thus, a first draft of inductive categories was developed and refined alongside the interviews.

All interviews were recorded and automatically transcribed. While data saturation was reached after 12 participants, a total of 14 interviews were conducted (7 per university) to satisfy the study’s preapproved sampling protocol.

### Data Analysis

The researcher who conducted the interviews also reviewed the automatic transcription against the video recordings; spelling errors and unclear words were corrected. The profiles of the interviewees were saved separately, and all data were anonymized. Each interviewee’s name was replaced with “Interviewee” plus a randomly assigned number and the abbreviation of the university (MUW for Medical University of Vienna and MUI for Medical University of Innsbruck). Text passages by the interviewer were marked with “Moderator.” To improve readability, repetitions and filler words (eg, “um” and “hm”) were removed.

For data management and analysis, the transcripts were loaded into the qualitative data analysis software ATLAS.ti for Windows (version 25.0.1; ATLAS.ti Scientific Software Development GmbH). The anonymized transcripts were reread, and the inductive categories were developed (Table S2 in Multimedia Appendix 2). Based on these categories, case summaries [24] were created for 3 randomly selected interviews. A second rater, not involved in the study, independently analyzed the interviews and developed additional case summaries. These summaries were compared, and discrepancies were discussed and resolved before the remaining case summaries were produced. Finally, all case summaries were consolidated and reorganized according to the established categories.

### Ethical Considerations

The ethics committee of the MUW deemed this study exempt from requiring a formal decision. Approval was granted by the data security board of the MUW. All participants received a formal invitation letter by email, including detailed information about the study purpose and a declaration of informed consent. Participation was voluntary.

## Results

### Qualitative Policy Framework Analysis for the Curriculum Change Process

To understand the policy framework of the universities for curriculum monitoring and change processes, the relevant regulations were analyzed. Austrian universities are regulated by the Universities Act (Universitätsgesetz [UG]) [28], the Higher Education Quality Assurance Act (Hochschul-Qualitätssicherungsgesetz) [29], and the Austrian University Development Plan (Gesamtösterreichischer Entwicklungsplan) [30]. Specifically, § 2 para 1 UG references Art 17 of the Basic Law on the General Rights of Citizens, which guarantees the “Freedom of sciences and teaching” [31]. According to the common interpretation, this freedom encompasses both the content to be taught and the manner of teaching. §14 UG [28] defines the need for a university internal quality management system and continuous evaluations, while § 2a (2) Hochschul-Qualitätssicherungsgesetz [29] emphasizes that teaching must comply with the current state of knowledge within each respective discipline. Although these requirements remain relatively broad, they structurally favor siloed curriculum models where educational content is primarily viewed from the perspective of individual subject areas.

Based on these documents, the medical universities developed their institutional frameworks. Overall, the MUW documents primarily focus on ensuring that scientific lecturers align their teaching with current scientific knowledge [32]. However, certain peripheral annotations suggest an awareness of structural challenges. The White Paper on Teaching at the MUW [33] cites an external audit report recommending “Increased coordination of curriculum content and learning objectives.” The MUI emphasizes within their documents “Curricula are continuously updated and adapted to current development” [34,35]. They acknowledge “Intensity and speed of knowledge acquisition will continue to increase*”* and that “Innovation will be much more strongly orientated towards the boundaries between the established disciplines than before*”* [36].

Consequently, the institutional documents reveal distinct strategic orientations between the institutions. The MUI states its commitment to a proactive approach by regularly monitoring curricular content to adapt to rapidly changing knowledge and to strengthen intracurricular alignment [35]. The MUW follows a more reactive approach that relies on students and lecturer evaluations while focusing on the knowledge development within specific medical specialties [37].

Overall, the policy framework provides relatively loose guidelines for the universities. While it mandates a quality management system, it specifies neither the relevant topics and key performance indicators nor the concrete curricular renewal processes. Crucially, this lack of a rigid top-down prescription presents a distinct opportunity for universities, granting them the institutional autonomy required to develop their frameworks toward an agile curriculum change process. However, despite this flexibility, the institutional frameworks of universities remain predominantly focused on specialty-specific improvements, treating general curricular adaptability merely as a secondary consideration. These policy findings serve as background information for the development of the interview guide.

### Qualitative Study of the Curriculum Change Process

The interviews were conducted between March and July 2025 and lasted an average of 53:45 (SD 14:37) minutes. The participants included 7 participants from the MUW and 7 participants from the MUI. Seven participants identified as female and 7 as male. Twelve participants held a habilitation (the postdoctoral lecture qualification “venia legendi”), and 2 were medical residents. The participants were also balanced according to the following characteristics: university governing body versus boards for curriculum operations and preclinical teaching versus clinical teaching. To ensure anonymity, the profiles were not described in more detail. The key themes were identified and summarized as follows: society’s evolving needs for the health care system, impact on the doctor-patient relationship, curriculum governance and adaptation process, dynamics and challenges of curriculum adaptation, and current usage of learning objectives.

### Society’s Evolving Needs for the Health Care System

#### Impact of Policies on Health Care Delivery

In 2023, the health care policy “digital before outpatient before inpatient” [38] was introduced as a strategic directive for the Austrian health care system. To date, the Austrian health care system does not have a formal gatekeeping mechanism. This means patients can freely choose whether they want to see a general practitioner, a specialist, or a hospital directly. The introduction of “digital before outpatient before inpatient” is not just a step toward a digital patient journey; it is also a first step toward a gatekeeper system.

*So, I believe that this is also a re-education process somewhere in society, that you cannot just go to the university hospital and then get everything, but that there are a few places beforehand*.

[Interviewee3_MUI]

All participants were positive about the new strategy; they acknowledged that the implementation of digital first will reduce the waiting time for patients and improve the management of patient flow for specialists and hospitals. However, some participants noted that they missed a detailed implementation plan.

#### Impact of Policies on Medical Education

The introduction of the policy “digital before outpatient before inpatient” will have implications for doctors and for medical education. Patients will be much more efficiently triaged to the right specialist and health care level. There will be a need to use telemedicine, digital monitoring, remote patient management, and integrated health care platforms. The data from the nationwide electronic health record system, which covers 97% of the population and is currently viewed primarily as a data repository, will enable a holistic approach to treatment.

*It will also have an impact on the job profile; I am one hundred per cent sure of that. In my opinion, we do not have it in the curriculum yet*.

[Interviewee3_MUI]

Most participants confirmed that this will have an impact on the medical profession but also recognized that it is not yet considered in current medical training.

### Impact on the Doctor-Patient Relationship

#### Changing Communication Needs

Participants were unanimously sure that digitalization will influence communication between doctor and patient. They mentioned that physical contact will decrease and that the importance of dialogue will increase. Some participants were concerned that reduced physical contact could negatively affect the doctor-patient relationship.

*As there is less contact, it becomes more impersonal. As there is less time available, there may be less trust between doctor and patient*.

[Interviewee7_MUI]

Some participants raised concerns regarding attempts to replace interpersonal medical interaction for patients with mental illnesses with digital triage systems or algorithmic symptom assessments. A few participants mentioned that digitalization would help to visualize medical diagnoses and improve the quality of communication.

*If artificial intelligence can create a visualization for me …, then I can have a much better, more focused conversation*.

[Interviewee4_MUW]

Most participants emphasized that, due to reduced physical contact with patients, communication skills are becoming even more important for doctors. Furthermore, patients will confront doctors with information from internet search engines, artificial intelligence (AI) tools, and TikTok influencers, which makes communication even more challenging.

*Doctors will be confronted with many more questions, as patients have already formed an opinion via Google and ChatGPT. This will take up more time again. This will change medical communication; medical students need to be prepared for this to be a positive thing*.

[Interviewee6_MUW]

Several participants discussed the growing role of digital communication channels such as video consultations, messaging systems, and remote consultations. While these tools were considered useful supplements, they stressed that digital communication cannot fully replace direct physician-patient interaction. At the same time, some participants described increasing communication challenges, including unrealistic expectations regarding physician availability, immediate digital responses, and constant accessibility.

About half of the participants mentioned that they are afraid that current students are too focused on diagnostic tools and are less capable of conducting physical examinations. This concern was consistent with the general view that the teaching of existing core medical competencies must be ensured.

*The biggest challenge for doctors will be not to lose touch with the patient. I think patient-orientation and working with patients is what students are least familiar with. The students are already very apparatus-orientated and use multidisciplinary clarification at an early stage before they even speak to the patient*.

[Interviewee11_MUI]

#### Impact of Telemonitoring and Wearables on the Doctor-Patient Relationship

Most participants highlighted the potential of patient monitoring and the resulting diverse communication needs. This includes the use of data from medically certified applications, data from nonmedically certified applications, and digitally implemented questionnaires (eg, quality-of-life questionnaires).

*… patients do not send the data from their wearables to the software provider, but to their doctor, who then proactively recognizes changes and calls the patient in or makes a video consultation*.

[Interviewee6_MUW]

These tools were perceived as particularly useful for chronic disease management, preventive medicine, and long-term follow-up care, provided that the collected data are discussed with the patient to initiate clinical or behavioral changes.

#### Impact on the Administrative Burden for Doctors

One of the strongest consensual themes concerned the expectation that digitalization should reduce administrative workload. All participants repeatedly criticized inefficient documentation systems, duplicate data entry, fragmented hospital information systems, and excessive bureaucracy.

*That preliminary results and questionnaires can be uploaded by the patient and automatically summarized in preparation for the first appointment*.

[Interviewee6_MUW]

*We do the patient information by phone; a doctor sits there and asks the questions and listens to the YES or NO. You could also clarify the information with a good video*.

[Interviewee2_MUW]

Participants observed many options, such as uploading preliminary diagnostic findings via the internet, and emphasized the capabilities of chatbots to perform anamneses, video-supported patient information, coding of diagnoses, and the preparation of doctor’s letters. For internal processes, the participants hoped for a seamless integration of the different platforms.

### Curriculum Governance and Adaptation Process

#### Description of Current Curriculum Change Processes

The curriculum of both universities follows an integrated approach [39]. The organization around the development and operations of the curriculum differs. The MUW participants described that the governing body consists of the rectorate and the curriculum commission, a board of the senate. In addition, the Curriculum Directorate has a strategic operational focus, as also described in the statutes (section III, para 4 (1) 2) [40]. This includes “creating guidelines for coordinating curriculum content and specifying the learning and training objectives of each curriculum based on suggestions from curriculum coordinators,” whereby curriculum coordinators coordinate a specific curriculum module. Most participants described the curriculum adaptation processes as highly complex and politically negotiated. There is a common agreement that changes are first discussed in the Curriculum Directorate, where proposals for changes are prepared. These are then submitted to the Vice-Rectorate for Teaching and the Rectorate. Finally, the Senate votes on the changes and approves them. Some participants stated the Vice-Rectorate for Teaching can intervene, but this is always seen as invasive and aggressive.

The MUI participants stated that they do not have a Curriculum Directorate. Instead, their Medical Program Directorate focuses on monitoring educational quality and introducing recommendations for future curriculum optimization. As a result, the Curriculum Commission and the Rectorate are much more closely involved in the operational strategic development of the curriculum. All MUI participants agreed that the Vice-Rectorate for Teaching very often develops new initiatives. For major structural changes, the Curriculum Commission determines which subjects are included at the degree program level. Additionally, some participants mentioned that a special educational advisory board, consisting of international experts, has been established to strengthen strategic capabilities.

#### Initiators of Curriculum Changes

Most MUW participants described that at MUW, great emphasis is placed on “freedom of teaching,” and successful curriculum innovation often depends on motivated individuals rather than systematic institutional processes. Unfortunately, this bottom-up approach also leads to several participants emphasizing a lack of central strategic coordination.

*Changes to the curriculum are primarily initiated by teachers and tertial or module coordinators, who try to keep improving the curriculum*.

[Interviewee12_MUW]

If new subjects emerge, there will be an extensive discussion about the reallocation of curriculum space. Some participants stated that the Curriculum Directorate will coordinate the discussion, and influence from the governing body will be seen as intrusive. This decision process can last 1 to 2 years.

Most MUI participants described that the Vice-Rectorate for Teaching very often initiates curriculum changes and cooperates intensively with the Curriculum Commission. The resulting top-down approach is appreciated by survey participants. Thereby, the Curriculum Commission determines which subjects are included at the degree program level and, together with the Vice-Rectorate of Teaching, also recommends the redistribution of curriculum hours.

This is also reflected in participants’ statements:

*There is very good cooperation between the Vice-Rector for Teaching and the Curriculum Commission. Many of the activities we undertake originate from the Vice-Rector for Teaching*.

[Interviewee9_MUI]

*When it comes to redistributing hours, it would really require changes from above*.

[Interviewee11_MUI]

#### Aligning New Content Within the Existing Curriculum

Most participants from both universities indicated that new and emerging technologies within their specialty are identified by the module coordinator and lecturers and implemented into the existing curriculum space. The module coordinator ensures that the content from the different lecturers is aligned within that module. However, all participants agreed that there is no formalized intermodule coordination process in place to ensure alignment between the various modules. It is up to the personal interests of the specific lecturer to check the slides of adjacent lectures.

*Well, I have never experienced anyone telling me to look. If I do not sit down and look to see if there are slides from other lecturers, then it will not happen*.

[Interviewee6_MUW]

Especially, the MUW participants mentioned that it would be difficult to discuss overlaps, gaps, or outdated content with lecturers from other modules. Some MUI participants added that there is an open end-of-semester meeting with students to discuss whether they experienced any overlap, gap, or outdated content. The findings suggest that the comprehensive longitudinal integration of digitalization remains organizationally difficult.

### Dynamics and Challenges of Curriculum Adaptation

#### Need to Include Digitalization Within the Curriculum

All participants agreed that digitalization should be part of the medical curriculum, but they noted that the current curriculum insufficiently prepares medical students for the digital transformation. Most participants also repeatedly observed that students are often less digitally competent than assumed, particularly regarding critical AI literacy. Many participants view elective courses as a starting point but favor longitudinal implementation across all specialties.

All participants also agreed that, for undergraduate students, the focus should be on learning how to use current digital applications in conjunction with critical thinking to distinguish reliable from unreliable information. Students should also develop a basic understanding of statistics and the meaning of “data” to better understand the strengths and weaknesses of digital tools. In this context, some participants mentioned that the underlying data for digital tools are not always adapted to the local population profile and local medical guidelines.

The participants also unanimously agreed that IT basics, working with big data, data management, and pattern recognition with AI should be trained in postgraduate and specialty-specific courses.

The main consensus among participants was that the medical curriculum needs to ensure that doctors have a broad overview of digital tools, can advise patients on finding the right tool, and can explain the results and their implications for further treatment.

*If the patient comes to the doctor and the doctor says: I am sorry, I have never seen that before, then it will not go down well. And if he has no idea about digital tools, then he will not be able to use them for treatment*.

[Interviewee10_MUW]

#### Challenges for Curriculum Changes

Several participants explained that the formal curriculum primarily regulates structural aspects such as the number of teaching hours, module organization, assessment structures, and semester distribution. Most participants recognized that the process of change within the curriculum is slow. At the MUW, the reprioritization of existing curriculum subjects emerges as the most prominent stumbling block. There are no clear processes for reprioritizing curriculum content; instead, it is a matter of give and take. As a result, newly emerging subjects often lead to long-lasting political discussions. At the MUI, the reprioritization process is guided by the Vice-Rector for Teaching and the Curriculum Commission and is therefore more efficient. Several participants from both universities criticized the absence of systematic longitudinal coordination across modules and semesters.

There is a general tendency across several participants to believe that curriculum changes do not necessarily have to follow the state of digital development quickly; rather, it makes sense to wait until the development stabilizes.

*I do not believe that curriculum changes necessarily have to follow the state of digital development quickly. Things change so quickly in the digital world that it makes sense to take a little time to see where the journey is really going. A few things sprinkled in at the beginning certainly cannot hurt. I think the curriculum is flexible enough for that*.

[Interviewee11_MUI]

This approach of waiting for robust evidence also delays the implementation of rapidly evolving knowledge. In society, this is perceived as a desperate clinging to old ways of behaving, which directly contradicts the previously stated requirement that doctors need to be able to advise the patient on the appropriate tool for treatment or prevention.

### Current Usage of Learning Objectives

#### Formal Use of Learning Objectives

All participants confirmed that both universities have formally established learning objectives as a framework for the curriculum. Learning objectives are required for the approval of a new lecture but remain an inconsistently operationalized tool. Some participants view them more as a bureaucratic necessity and questioned whether learning objectives are systematically reviewed, updated, or evaluated over time.

*For the main lectures, the learning objectives are kept relatively general. We have not spelled them out in detail. It is probably quite reasonable to reconsider what the average student should know*.

[Interviewee3_MUI]

In this context, several participants referred to the evaluation process. They reported that evaluations focus primarily on organizational and didactic aspects rather than the actual alignment between teaching activities and intended learning outcomes. The findings suggest that learning objectives often function as formal accreditation requirements rather than dynamic curriculum–steering instruments.

#### Learning Objectives as Curriculum Monitoring and Coordination Tools

All participants unanimously described deficits in systematic, curriculum-wide coordination of learning objectives. Neither university uses learning objectives to monitor the curriculum or to provide a holistic overview. Consequently, coordination between modules frequently depends on informal communication and individual initiative. As a result, several participants highlighted redundancies across modules, missing competencies, and a lack of monitoring regarding the longitudinal progression of content.

*There is no evaluation and monitoring of the curriculum in terms of learning objectives and integrative character*. [Interviewee8_MUW]

*Learning objectives are an absolute must for us; they are always present. How they are monitored is a good question. I do not know*.

[Interviewee1_MUI]

Several participants view central, learning objective–based monitoring as an opportunity to elevate discussions to a more substantive level and to achieve faster curriculum adaptation. At the same time, participants noted tensions between standardization and academic freedom. Some participants expressed concerns that stronger central regulation of learning objectives might restrict lecturers’ autonomy. Overall, the findings indicate that learning objectives are currently not utilized as active curriculum monitoring and coordination instruments.

## Discussion

### Findings

Digitalization in medicine is a relatively new field that has not emerged from traditional fields of medicine but rather from society’s need to streamline processes in the health care system and optimize health care spending. Furthermore, it does not affect only one medical specialty but is a cross-cutting technology. The implementation of such cross-cutting and fast-evolving content challenges existing decision-making and implementation processes in medical universities. Structures originally designed to accommodate the gradual growth of specialty-specific knowledge are increasingly overwhelmed by the influx of new, externally driven developments [41].

This study uses digitalization in medicine as an example to examine how efficiently existing processes for curriculum change and renewal can cope with the current pace of change in technology, information and data dissemination, and health care. To determine the current status, the results of a qualitative study with faculty members at 2 Austrian medical universities were compared with the curriculum development frameworks of Kern [5] and Harden [6]. Both frameworks stress the importance of clearly defined educational goals, learning objectives, and learning outcomes to hold medical educators accountable and to provide inputs for reforming and managing medical education [5,23]. Both also stress the importance of monitoring: monitoring the existing curriculum, as well as monitoring the continuous development of medical science, technology, and society’s needs. Kern [5] uses learning objectives to assess whether students achieve the expected outcomes, whereas Harden [7] organizes learning outcomes into curriculum maps to identify overlaps and gaps.

Study participants acknowledged the significant impact of digitalization on the medical profession. In addition to new tools that will change the way doctors do their work, digitalization is expected to transform health care delivery and the doctor-patient relationship. However, participants also agreed that these changes are not yet systematically reflected in the current curriculum.

The universities examined mainly rely on processes tailored to gradual, discipline-specific curriculum development. MUW’s bottom-up approach encourages incremental improvements and aligns well with past periods of slow curricular change. Hamsal [42] described this organizational behavior as the Icarus Paradox. In addition, MUW places a high value on teaching autonomy. This emphasis on academic freedom inadvertently reinforces resistance to reform, directly enabling the phenomenon of “curriculum hoarding” described by Romanelli [43] (see also Bearman et al [44]). In contrast, MUI applies a combined top-down and bottom-up approach, allowing for somewhat more flexible responses to external developments. Nevertheless, both universities depend primarily on external triggers for curricular change rather than on systematic internal review mechanisms.

Existing curriculum monitoring is established with a focus on student satisfaction and their learning experiences. While such evaluations fulfill formal requirements [28], they do not provide sufficient data to improve the organization of curricular content or the overall curriculum structure. Participants reported that learning objectives are mandatory for the approval of new modules; however, no procedures exist to monitor them thereafter. Although both universities originally developed integrated curricula and adopted learning objectives as a guiding principle, the lack of structured monitoring has led to growing gaps and overlaps over time. In slowly evolving environments, such shifts may pose limited issues. However, in fields characterized by rapid innovation, these inconsistencies can lead to curriculum fragmentation, as described by Harden [22], and to integrated modules without an integrated curriculum.

Although some module coordinators actively use learning objectives within their responsibilities, participants agreed that the absence of formal intermodule coordination contributes to divergence between modules. Without consistently monitoring learning objectives and learning outcomes, the university relies solely on student assessments to verify whether overarching educational goals are achieved. Consequently, curriculum mapping [7], which would make learning objectives and their interdependencies visible across the curriculum, is not implemented at the universities examined. Instead of a structured, evidence-informed discussion guided by shared priorities, the integration of new cross-cutting technologies, such as digitalization, becomes an exhausting negotiation for space in a packed curriculum.

In his work “The Integration Ladder,” Harden [22] discusses the importance of making interdependencies between modules visible and understandable. Visualizing these interdependencies helps to maintain curricular coherence and ensures that content is aligned with the overall educational goals. It also clarifies which modules need to be informed or adapted when changes are introduced elsewhere in the curriculum. Existing models illustrate potential approaches: for example, Theurich et al [45] compared the learning objectives of their curriculum against a national catalog to validate curricular relevance. Naylor and Torres [46] used student surveys to assess the implementation of learning objectives in 2 courses. In contrast, participants from both universities reported intensive intramodule coordination but no formal process for inter-module coordination. Participants are even reluctant to approach colleagues, as this could be perceived as intrusive.

This study shows that curriculum processes suitable for slow-evolving, specialty-specific development are not able to keep pace with today’s changes in technology, information and data dissemination, and health care delivery [47]. This mismatch generates a widening gap between current medical training and the competencies required by future health care systems. To reduce this gap, medical universities need to transform their curriculum governance and operational organization [48-51]. This transformation needs to address the tension between academic freedom and coordinated curriculum development [52]. While lecturers should maintain autonomy over teaching methods and disciplinary depth, aligning their content with overarching program competencies must become a centralized function. Therefore, the study indicates that modern curriculum governance must balance decentralized expertise with centralized coordination mechanisms capable of maintaining curricular coherence.

To operationalize this balance, the Kern needs-based curriculum development concept and the Harden systematic curriculum monitoring and integration concept provide a solid theoretical foundation. Building on these models, institutions can draw on established change management frameworks tailored to medical education. For instance, Gale and Grant (AMEE Medical Education Guide Number 10) [53] provide structured guidelines for organizational change in a medical context, while Odiaga et al [51] demonstrate how the Kotter change model can successfully drive the required cultural transformation. Implementing these frameworks requires raising curriculum planning quality by providing faculty development on learning objectives, curriculum integration, and curriculum monitoring and mapping. As a result, the curriculum development process will move from reactive, module-centered change toward proactive, program-level governance.

Regulatory bodies can further support this transition by requiring continuous, forward-looking monitoring of learning objectives and educational goals to meet the needs of society, patients, medical students, and health care providers in the 21st century.

### Limitations

This study evaluates existing curriculum processes by mapping them onto the frameworks of Kern and Harden. Since the analysis is grounded in these specific frameworks, the application of alternative frameworks might reveal different perspectives on the identified challenges. Although the results represent a snapshot of curriculum processes at only 2 of the 4 Austrian public medical universities, the findings may not be fully transferable to medical universities operating under different legal, cultural, or accreditation frameworks.

Participant recruitment followed a snowball sampling approach, where senior key individuals nominated additional participants. This strategy may have introduced selection bias. Furthermore, as attitudes toward new technologies often vary with age, a potential age-related bias among participants cannot be excluded.

Because this study required in-depth knowledge of curriculum change processes, other stakeholders, such as students, policymakers, and administrative staff, were excluded. While these groups were omitted due to the study’s specific focus, customized research targeting these cohorts could provide a valuable external perspective on curriculum change processes.

Finally, this study did not include an analysis of existing documented curriculum processes, teaching materials, or learning outcomes. Consequently, no conclusions can be drawn regarding the actual impact of the observed curricular structures and processes on student learning.

### Conclusions

In this study, digitalization in medicine serves as an example of how existing curriculum processes are challenged by emerging topics. The challenge lies not primarily in the rapid addition of new content but in the development of agile, integrated processes that support strategically coordinated curriculum design and enable timely, future-oriented adaptation. Curriculum management driven predominantly by operational considerations risks adding new content without systematically removing less relevant material. Integrating established curriculum development frameworks into curriculum management processes can support prioritization and help maintain curricula that are both agile and future-oriented. As students already provide feedback via web-based evaluation, one simple step forward is to include the successful achievement of individual learning objectives within this evaluation. This approach would provide immediate feedback to lecturer and curriculum coordinators.

Learning objectives, in particular, provide a powerful tool for tracking curriculum content, coordinating modules, and embedding cross-cutting topics such as digitalization throughout the curriculum. In addition, strengthening mechanisms for cross-module coordination and systematic curriculum monitoring is crucial. Through such approaches, medical education can ensure that graduates are not only familiar with digital tools but also resilient, critical, and adaptable to continuous change. While governmental initiatives strongly support the development of digital skills in general, more comprehensive and legally binding guidelines for agile curriculum processes could further facilitate the integration of rapidly evolving knowledge into medical education.

Even though the study was conducted in Austria and the specific governance structures may differ across countries, the underlying challenge of integrating fast-moving knowledge into traditionally slow-changing curricula appears universal. In highly regulated systems, curriculum adaptation may be constrained by accreditation standards or national competency frameworks. In less regulated or resource-constrained systems, challenges may include limited digital infrastructure, faculty expertise, or institutional capacity. Here, structured learning objectives provide relatively low-cost mechanisms for alignment, prioritization, and continuous adaptation.

This study contributes to the growing body of knowledge on curriculum development and renewal and highlights areas for future research. Further studies should include qualitative interviews with curriculum planners to explore challenges in implementing curriculum renewal in greater depth. This research aims to provide curriculum planners with a practical toolbox for the efficient and effective management of curriculum processes, enabling even resource-limited medical degree programs to adopt a future-oriented approach. Ultimately, this field of research is essential for a holistic understanding of medical curricula and for strengthening the shared sense of responsibility among medical educators.

## Supplementary material

10.2196/96244 Multimedia Appendix 1 Analyzed university regulations.

10.2196/96244 Multimedia Appendix 2 Deductive and inductive grading categories.

## Abbreviations

AI: artificial intelligence
COREQ: Consolidated Criteria for Reporting Qualitative Research
MUI: Medical University of Innsbruck
MUW: Medical University of Vienna
UG: Universitätsgesetz
