# Supplementary material for: Systemic Barriers to Curriculum Adaptation for Rapidly Changing Knowledge in Medical Education: Qualitative Study
Source: JMIR Med Educ. 2026 Jul 15;12:e96244. doi: 10.2196/96244 (PMC13372216; doi:10.2196/96244)
Supplement: Multimedia Appendix 1 [file mededu-v12-e96244-s001.docx]

| general | University Act (Universitätsgesetz - UG)  Higher Education Quality Assurance Act (Hochschul-Qualitätssicherungsgesetz – HS-QSG)  Austrian University Development Plan (Gesamtösterreichischer Entwicklungsplan 2025-2030) |
| --- | --- |
| Medical University of Vienna | Satzung, Studienjahr 2024/2025  Entwicklungsplan 2025-2030  Leistungsvereinbarung 2025-2027  Wissensbilanz 2023  Curriculum der Humanmedizin June 2022  Whitepaper Lehre  Auditbericht der AHPGS Akkreditierung gGmbH 2021 |
| Medical University of Innsbruck | Satzung Teil Evaluation, Studienjahr 2009/2010  Entwicklungsplan 2025-2030  Leistungsvereinbarung 2025-2027  Wissensbilanz der Medizinischen Universität Innsbruck 2024  Curriculum der Humanmedizin Studienjahr 2024/2025  Ergebnisbericht der Agentur für Qualitätssicherung und Akkreditierung Austria 2024 |
