# Supplementary material for: Systemic Barriers to Curriculum Adaptation for Rapidly Changing Knowledge in Medical Education: Qualitative Study
Source: JMIR Med Educ. 2026 Jul 15;12:e96244. doi: 10.2196/96244 (PMC13372216; doi:10.2196/96244)
Supplement: Multimedia Appendix 2 [file mededu-v12-e96244-s002.docx]

| Society's evolving needs for the healthcare system | |
| --- | --- |
|  | Impact of policies on healthcare delivery |
|  | Impact of policies on medical education |
|  | Evolving role of doctors in a digitalized healthcare system |
|  | Digitalization as unavoidable transformation |
| Impact of digitalization on doctor-patient relationship | |
|  | Changing communication needs |
|  | Impact on telemonitoring and wearables on doctor-patient relationship |
|  | Impact on the administrative burden for doctors |
|  | Patients’ acceptance of digital medicine |
|  | Pretrained patients (Internet, Google, AI) create more complex discussions |
|  | Importance of bedside medicine |
|  | New roles of doctors (e.g. health navigator) |
|  | Risk of overreliance on artificial intelligence generated diagnosis |
|  | Handling of irrelevant automated diagnosis |
| Curriculum governance and adaptation process | |
|  | Description of current curriculum change processes |
|  | Initiator of curriculum changes |
|  | Aligning new content within the existing curriculum |
|  | Curriculum changes depending on individual initiatives |
|  | Lack of inter-module monitoring processes |
|  | Shift in student attendance away from lecture halls since the onset of COVID-19 |
|  | Collection and impact of student feedback |
| Dynamics and challenges of curriculum adaptation | |
|  | Need to include digitalization within the curriculum |
|  | Challenges for curriculum changes |
|  | Evaluations focus primarily on organizational and didactic aspects |
|  | Number of lecture hours as an indicator of a department's importance |
|  | Sometimes it's best to wait until the professor retires |
|  | Decision processes are to slow |
|  | Competition for limited curriculum time between departments |
| Newly Emerged Main Category: Current usage of learning objectives | |
|  | Formal use of learning objectives |
|  | Learning objectives as curriculum monitoring and coordination tool |
|  | Regular reevaluation is missing |
|  | Weak or non-existing coordination across different modules |
|  | Academic freedom versus curriculum monitoring and coordination |
|  | Students' focus on memorizing past exam questions |
|  | Student learning strategies with external commercial tool (e.g. Amboss) |
